# Anti-Inflammatory and Analgesic Effects of the Marine-Derived Compound Excavatolide B Isolated from the Culture-Type Formosan Gorgonian *Briareum excavatum*

**DOI:** 10.3390/md13052559

**Published:** 2015-04-27

**Authors:** Yen-You Lin, Sung-Chun Lin, Chien-Wei Feng, Pei-Chin Chen, Yin-Di Su, Chi-Min Li, San-Nan Yang, Yen-Hsuan Jean, Ping-Jyun Sung, Chang-Yih Duh, Zhi-Hong Wen

**Affiliations:** 1 Department of Marine Biotechnology and Resources, Asia-Pacific Ocean Research Center, National Sun Yat-sen University, Kaohsiung 80424, Taiwan; E-Mails: chas6119@gmail.com (Y.-Y.L.); gobetter04@yahoo.com.tw (Y.-D.S.); afred75910@gmail.com (C.-M.L.); 2 Department of Orthopaedic Surgery, Ping-Tung Christian Hospital, Ping-Tung 90059, Taiwan; E-Mails: linsungchun@yahoo.com.tw (S.-C.L.); jean.tang@msa.hinet.net (Y.-H.J.); 3 Doctoral Degree Program in Marine Biotechnology, National Sun Yat-sen University and Academia Sinica, Kaohsiung 80424, Taiwan; E-Mails: qscjuejuejue@gmail.com (C.-W.F.); peichin1128@gmail.com (P.-C.C.); 4 National Museum of Marine Biology & Aquarium, Pingtung 94450, Taiwan; E-Mail: pjsung@nmmba.gov.tw; 5 School of Medicine, College of Medicine and Department of Pediatrics, E-DA Hospital, I-Shou University, Kaohsiung 84001, Taiwan; E-Mail: y520729@gmail.com; 6 Graduate Institute of Marine Biology, National Dong Hwa University, Pingtung 94450, Taiwan

**Keywords:** excavatolide B, carrageenan, lipopolysaccharide, inducible nitric oxide synthase (iNOS), cycloxygenase-2 (COX-2), anti-inflammatory

## Abstract

In recent years, several marine-derived compounds have been clinically evaluated. Diterpenes are secondary metabolites from soft coral that exhibit anti-inflammatory, anti-tumor and cytotoxic activities. In the present study, we isolated a natural diterpene product, excavatolide B, from cultured Formosan gorgonian *Briareum excavatum* and investigated its anti-inflammatory activities. We found that excavatolide B significantly inhibited the mRNA expression of the proinflammatory mediators, inducible nitric oxide synthase (iNOS) and cyclooxygenase-2 (COX-2), in lipopolysaccharide (LPS)-challenged murine macrophages (RAW 264.7). We also examined the anti-inflammatory and anti-nociceptive effects of excavatolide B on intraplantar carrageenan-induced inflammatory responses. Excavatolide B was found to significantly attenuate carrageenan-induced nociceptive behaviors, mechanical allodynia, thermal hyperalgesia, weight bearing deficits and paw edema. In addition, excavatolide B inhibited iNOS, as well as the infiltration of immune cells in carrageenan-induced inflammatory paw tissue.

## 1. Introduction

Several reviews have reported that marine organisms, such as sponges, soft corals, snails and seaweeds, are novel resources for drug discovery [1,2]. The secondary metabolites from these marine organisms have been shown to exhibit various bioactivities, and several of these natural products are now under preclinical and clinical trials [3]. Coral reefs are among the most productive marine ecosystems, and over 3000 new compounds have been discovered from corals in the past two decades alone [1,4,5]. Diterpenes from soft coral, such as cembrane and briarane, have been verified to show several bioactivities in previous chemical investigations [3,6,7]. Our previous reports also indicate that cembrane- or briarane-type diterpenes from soft coral could have anti-inflammatory potential, significantly inhibiting the expression of proinflammatory proteins, such as inducible nitric oxide synthase (iNOS) and cyclooxygenase (COX-2), in lipopolysaccharide (LPS)-stimulated murine RAW 264.7 macrophages [8,9,10,11].

Inflammation plays an important role in the development of many diseases, and different cell types are recruited, including monocytes/macrophages, neutrophils and lymphocytes, to the tissue during the process of inflammation [12,13,14]. The LPS-stimulated murine macrophage model is a well-known system to assess the anti-inflammatory activities of marine- and terrestrial-derived secondary metabolites *in vitro* [15,16,17,18]. LPS could upregulate the production of various pro-inflammatory mediators, including cytokines, chemokines and enzymes, such as COX-2 and iNOS, which play critical roles in inflammatory processes via activation of the nuclear factor (NF)-κB signaling pathway [19,20]. iNOS can produce nitric oxide to increase blood flow and vascular permeability, leading to the recruitment of infiltrating cells into inflamed tissue throughout the process of inflammation [21,22,23]. Moreover, overproduced NO reacts with superoxide anion (O^2−^), which causes tissue damage via oxidation [24]. NO can also upregulate COX-2 expression through the NF-κB signaling pathway [25,26]. COX-2 leads to prostaglandin (PGs) synthesis, which can upregulate the inflammatory response and enhance pain in chronic inflammatory diseases and pain disorders [27,28]. Our previous studies illustrate that soft coral-derived secondary metabolites can produce efficacious anti-inflammatory activity in *in vitro* and *in vivo* models with significant reductions in iNOS and COX-2 expression [9,29,30]. Thus, screening for inhibitors of the pro-inflammatory mediators iNOS and COX-2 in marine natural products is a promising avenue for drug development.

The briarane-type diterpene excavatolide B was first isolated from *Briareum excavatum* by Sheu *et al.* (1998) and shown to have a low cytotoxicity [31]. Wei *et al.* (2011) found that excavatolide B significantly attenuated 12-*O*-tetradecanoylphorbol-13-acetate (TPA)-induced skin inflammation in mice [32]. The carrageenan-injected paw in rat is a well-established animal model of inflammation for the evaluation of anti-inflammatory and analgesic compounds [29,30,33]. Several studies clearly indicate that the anti-inflammatory properties of compounds that inhibit iNOS or COX-2 expression in LPS-stimulated macrophages can attenuate inflammatory responses, swelling, pain and leukocyte infiltration in carrageenan-injected models [9,34,35,36]. In the present study, we aimed to illustrate the anti-inflammatory effects of excavatolide B, which was obtained from culture-type Formosan gorgonian *Briareum excavatum* from the National Museum Biology Aquarium (NMBA), in an LPS-stimulated murine macrophage model. We found that excavatolide B reduced iNOS and COX-2 mRNA expression and analgesic and anti-inflammatory activities of excavatolide B in a carrageenan-induced paw edema model.

## 2. Results

### 2.1. Cell Viability

**Figure 1:**
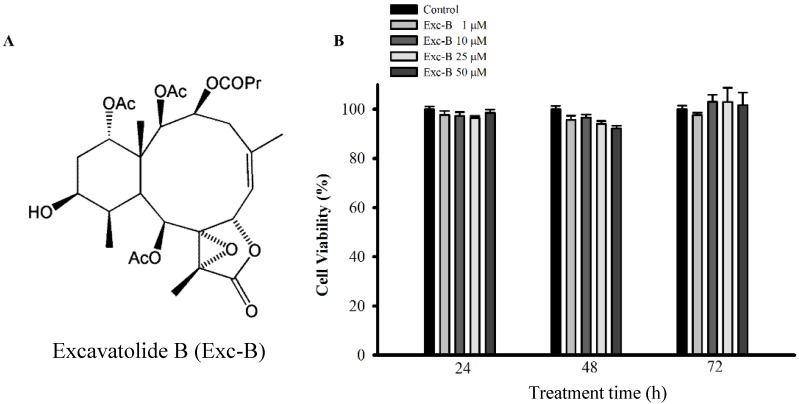
(**A**) Chemical structure of excavatolide B (Exc-B). The chemical structure of excavatolide B. Molecular formula, C_30_H_42_O_12_. Molecular weight, 595.6 Da; (**B**) Effect of excavatolide B on the viability of macrophage cells. Cell were incubated with different concentrations of excavatolide B for 24, 48 and 72 h, and cell viability was assessed by the Alamar Blue assay. Data from five independent experiments are presented as the mean ± SEM values.

To evaluate the effect of excavatolide B on the viability of RAW 264.7 macrophage cells, we used the Alamar Blue assay. The viability of macrophage cells at 24, 48 and 72 h after treatment with excavatolide B (1, 10, 25 and 50 μM) is shown in Figure 1B. Excavatolide B (1, 10, 25 and 50 μM) did not significantly affect the viability of macrophage cells 24, 48 and 72 h after treatment.

### 2.2. Effect of Excavatolide B on iNOS and COX-2 Gene and Protein Expression in LPS-Induced RAW 264.7 Cells

**Figure 2:**
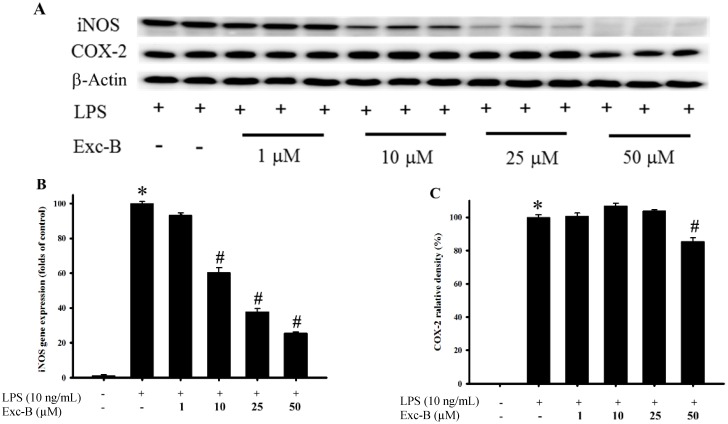

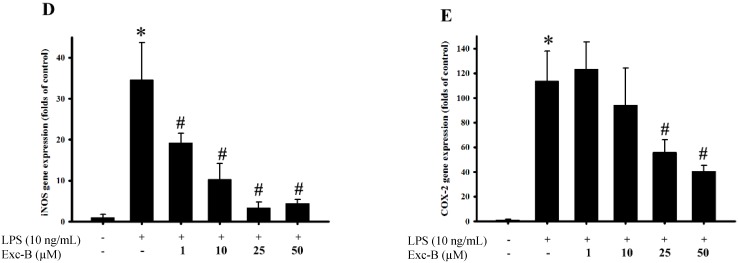
Effect of excavatolide B on inducible nitric oxide synthase (iNOS) and cyclooxygenase (COX)-2 mRNA and protein expression in lipopolysaccharide (LPS)-induced RAW 264.7 cells. RAW 264.7 cell were pre-treated with different concentrations of excavatolide B (1, 10, 25 and 50 μM) for 10 min before the LPS (10 ng/mL) challenge. (**A**) The Western blotting analysis corresponding to iNOS, COX-2 and β-actin protein from RAW 264.7 cells. The protein expression of iNOS (**B**) and COX-2 (**C**) was normalized by β-actin. The mRNA levels of iNOS (**D**) and COX-2 (**E**) were normalized by GAPDH. The data are shown as the mean ± SEM (*n* = 6). * *p* < 0.05 between LPS-alone compared with the control group. ^#^ *p* < 0.05 between LPS plus excavatolide B (1, 10, 25 and 50 μM) compared to the LPS treatment alone.

The effect of excavatolide B on iNOS and COX-2 gene and protein expression in LPS-induced RAW 264.7 cells is shown in Figure 2. The western blotting results demonstrate that the protein expression of iNOS and COX-2 was upregulated after stimulation with LPS for 18 h compared to the control group. Excavatolide B at doses of 1, 10, 25 and 50 μM shows significant dose-dependent inhibition of iNOS protein expression compared to the LPS-alone group. Excavatolide B (50 μM) also significantly inhibited COX-2 protein expression compared to the LPS-alone group. The qPCR results demonstrate that the gene expression of iNOS and COX-2 was upregulated after stimulation with LPS for 8 h compared to the control group. Excavatolide B at doses of 1, 10, 25 and 50 μM shows significant dose-dependent inhibition of iNOS gene expression compared to the LPS-alone group. Excavatolide B (25 and 50 μM) also significantly inhibited COX-2 gene expression compared to the LPS-alone group. Thus, excavatolide B demonstrated significant inhibition of LPS-induced iNOS and COX-2 gene and protein expression in RAW 264.7 murine macrophages.

### 2.3. Effect of Excavatolide B on Carrageenan-Induced Paw Edema in Rats

**Figure 3:**
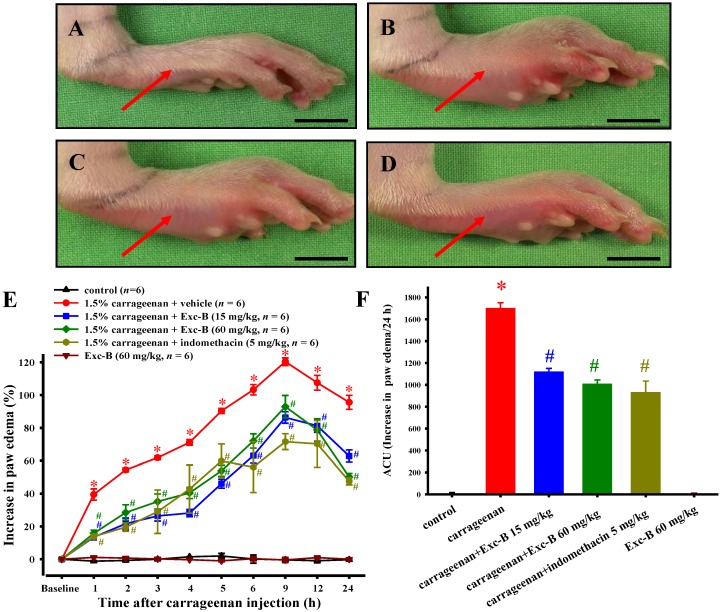
Effect of excavatolide B on carrageenan-induced paw edema in rats. Typical representative macroscopic photographs of paw from the (**A**) control, (**B**) 1.5% carrageenan + vehicle, (**C**) 1.5% carrageenan + 15 mg/kg excavatolide B and (**D**) 1.5% carrageenan + 60 mg/kg excavatolide B groups. The 1.5% carrageenan groups show obvious edema and redness (red arrow) on hind paws compared to the control group. The 1.5% carrageenan + excavatolide B (60 mg/kg) treatment could reduce edema and redness in carrageenan-induced paws in rats. Time courses of the anti-inflammatory effects of excavatolide B (15 or 60 mg/kg) and indomethacin (5 mg/kg) on carrageenan-induced rats, including paw edema (**E**) and the area under the curve (AUC) (**F**). Each time point or bar represents the mean ± SEM of six rats. The result for the different concentrations of excavatolide B were significantly different compared to the carrageenan group. A–D, scale bar = 1 cm. * *p* < 0.05 between carrageenan + vehicle group compared with the control group. ^#^ *p* < 0.05 between 1.5% carrageenan + excavatolide (15 or 60 mg/kg) groups compared with the 1.5% carrageenan + vehicle group.

Typical representative macroscopic photographs of the intraplantar injection of carrageenan into the right paw of rats are shown in Figure 3. The carrageenan group demonstrated edema and redness on the right paw (Figure 3B). Both 15 and 60 mg/kg of excavatolide B could effectively reduce edema and redness of hind paws after 100 μL 1.5% carrageenan injection (Figure 3C,D). Figure 3E illustrates the time-dependent increase of paw edema after carrageenan injection. Paw edema increased to approximately 120.38% ± 2.33% of baseline values 9 h after carrageenan injection in the carrageenan group. The 1.5% carrageenan + 15 or 60 mg/kg excavatolide B groups could significantly inhibit paw edema after 1–6, 9, 12 and 24 h compared to the carrageenan-alone group (Figure 3E). In the analysis of the AUC of the edematous effect-time curve, we found an overall dose-dependent effect of excavatolide B on carrageenan-induced paw edema. Low dose excavatolide B (15 mg/kg) also significantly attenuated carrageenan-induced paw edema, based on the AUC data. The results show that excavatolide B could effectively reduce paw edema with a dose-dependent effect in rats with carrageenan-induced inflammation.

### 2.4. Effect of Excavatolide B on Carrageenan-Induced Nociceptive Behaviors

**Figure 4:**
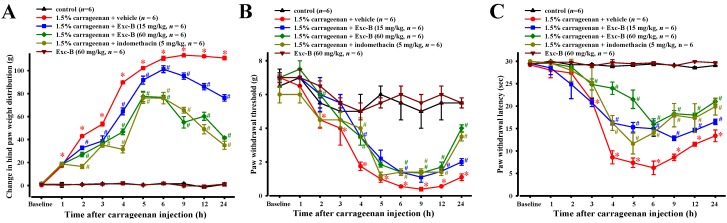
A time course showing the effects of excavatolide B on the carrageenan-induced pain behavior. Time courses of the analgesic effects of excavatolide B (15 or 60 mg/kg) on carrageenan-induced rats, including: mechanical allodynia (**A**); thermal hyperalgesia (**B**); and hind paw weight-bearing deficits (**C**). Each time point or bar represents the mean ± SEM of six rats. The results show that the different concentrations of excavatolide B and indomethacin (5 mg/kg) are significantly different compared to the carrageenan group. A–D, scale bar = 1 cm. * *p* < 0.05 between carrageenan + vehicle group compared with the same time points in the control group. ^#^ *p* < 0.05 between 1.5% carrageenan + excavatolide (15 or 60 mg/kg) groups compared with the 1.5% carrageenan + vehicle group.

No significant difference between the baseline values of all experimental groups were observed for paw withdrawal latency, paw withdrawal threshold or changes in hind paw weight distribution before carrageenan injection. Figure 4A shows the effect of excavatolide B on carrageenan-induced thermal hyperalgesia in the right hind paw. The paw withdrawal latency progressively decreased and reached a maximum response (6.24 ± 1.5 s) 6 h after carrageenan injection. Subcutaneous injection of excavatolide B (15 mg/kg) 1 h before carrageenan injection significantly inhibited thermal hyperalgesia 4–24 h later. Excavatolide B (60 mg/kg) also significantly inhibited thermal hyperalgesia 3–24 h after carrageenan injection. The results show that excavatolide B could dose-dependently reduce thermal hyperalgesia. Additionally, indomethacin (5 mg/kg) also significantly inhibited thermal hyperalgesia at 4 to 24 h after carrageenan injection. Figure 4B shows the effect of excavatolide B on carrageenan-induced mechanical allodynia hypersensitivity in paws. The paw withdrawal threshold progressively decreased and reached a maximum response (1.00 ± 0.39 g) 9 h after carrageenan injection. The carrageenan + 60 mg/kg excavatolide B group significantly inhibited mechanical allodynia hypersensitivity after 2, 4 and 6–24 h, and the carrageenan + 15 mg/kg excavatolide B group significantly inhibited the mechanical allodynia hypersensitivity at 4, 6, 12 and 24 h. Additionally, indomethacin (5 mg/kg) also significantly inhibited mechanical allodynia hypersensitivity at 4 and 6–24 h after carrageenan injection. Figure 4C shows the effect of excavatolide B on carrageenan-induced changes in hind paw weight distribution. The change in hind paw weight distribution progressively increased and reached a maximum value (113.5 ± 1.6 g) 9 h after carrageenan injection. Subcutaneous injection of excavatolide B (15 or 60 mg/kg) 1 h before carrageenan injection significantly reduced the change in hind paw weight distribution after 2–24 h. Additionally, indomethacin (5 mg/kg) also significantly reduced the change in hind paw weight distribution at 2–24 h after carrageenan injection. Moreover, the excavatolide B (60 mg/kg)-alone group did not show any pain and mortality in normal rats. Thus, the results show that excavatolide B could significantly reduce pain behavior in carrageenan-induced paws of rats.

### 2.5. Histological Analyses of Rat Paw Tissues

**Figure 5:**
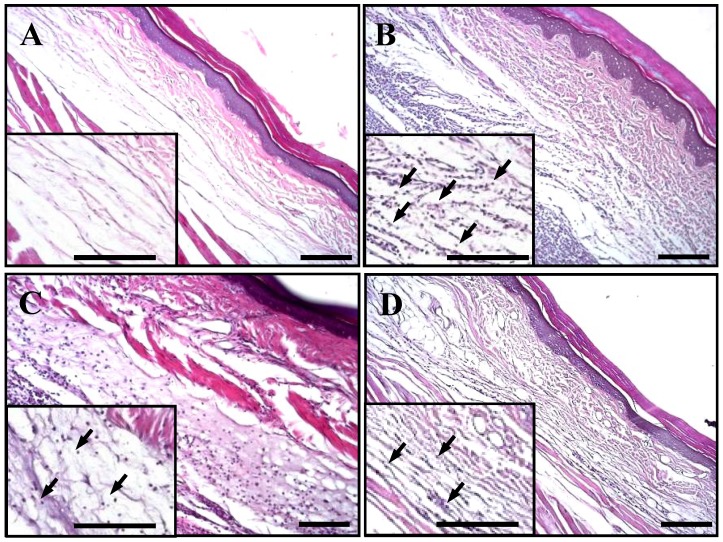

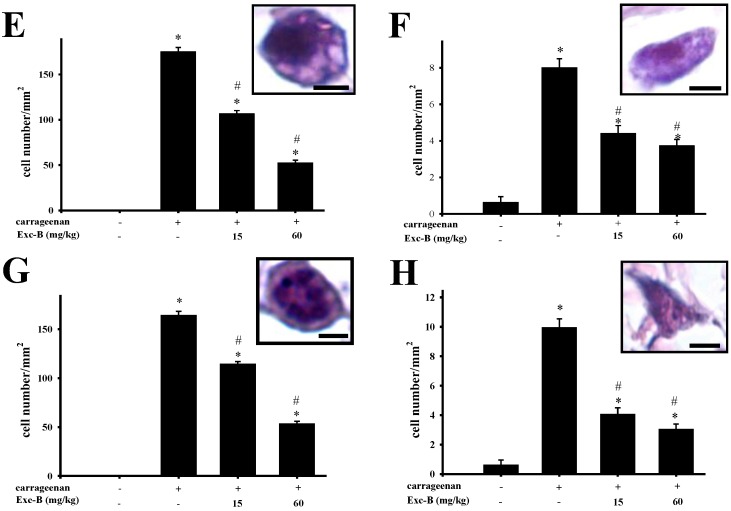
Histopathological assessment of the effect of excavatolide B on carrageenan-induced paw edema in rats. Representative sections of paw from the (**A**) control, (**B**) 1.5% carrageenan + vehicle, (**C**) 1.5% carrageenan + 15 mg/kg excavatolide B and (**D**) 1.5% carrageenan + 60 mg/kg excavatolide B groups stained with H&E stain. Upregulation of infiltrating cells (black arrow) was observed in paw sections from the 1.5% carrageenan group. Quantification of the number of monocytes (**E**), macrophages (**F**), neutrophils (**G**) and fibroblasts (**H**) in the paw tissue. Infiltrating cell numbers significantly decreased in the 1.5% carrageenan + excavatolide B (15 or 60 mg/kg) groups compared to the carrageenan group. * *p* < 0.05 compared to the control group. ^#^ *p* < 0.05 compared to the carrageenan group. A–D, scale bar = 100 μm; E–H, scale bar = 5 μm.

The anti-inflammatory effect of excavatolide B on carrageenan-induced paw edema in rats was histologically evaluated, and typical representative macroscopic photographs of the paw tissue sections from the control (Figure 5A), carrageenan + vehicle (Figure 5B), carrageenan + 15 mg/kg excavatolide B (Figure 5C) and carrageenan + 60 mg/kg excavatolide B groups (Figure 5D) stained with hematoxylin and eosin are shown. The control group showed normal paw tissue, and intraplantar injection of 1.5% carrageenan into the paw of rats displayed obvious accumulation of infiltrated inflammatory cells (Figure 5B) compared to the control group. However, the infiltrated inflammatory cells effectively reduced with excavatolide B (15 or 60 mg/kg) treatment 24 h after carrageenan injection. Neutrophils, macrophages, monocytes and synovial fibroblasts were quantified as shown in Figure 5E–H, and all cell numbers significantly increased in the carrageenan group compared to the control group. Excavatolide B (15 or 60 mg/kg) significantly and dose-dependently decreased the number of neutrophils, macrophages, monocytes and fibroblasts in carrageenan-injected paw tissue (Figure 4E–H).

### 2.6. Western Blot Analysis of iNOS Protein Expression in Rat Paw Tissue

**Figure 6:**
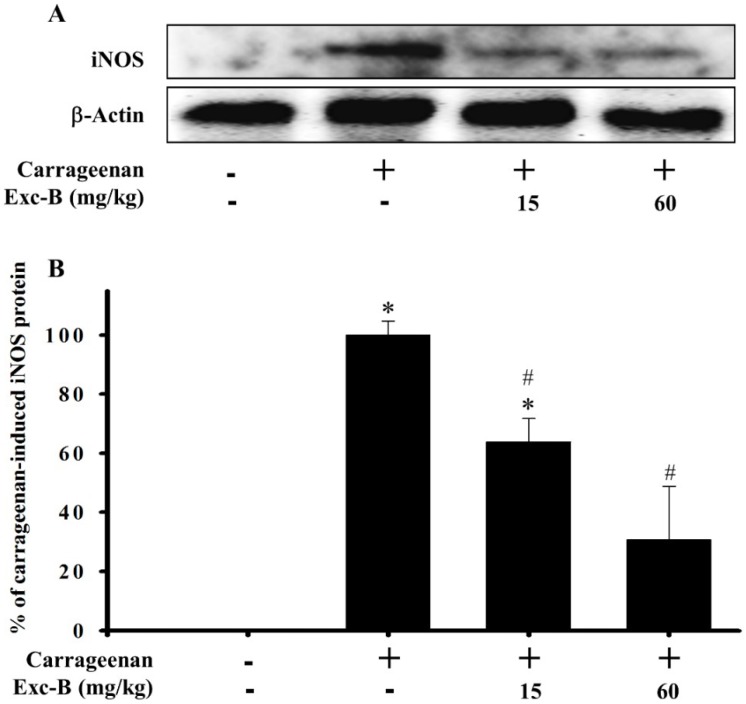
Effect of excavatolide B on inducible nitric oxide synthase (iNOS) protein expression in homogenized paw tissue from carrageenan-induced paw edema rats. Western blot analysis of the protein iNOS and β-actin (**A**). Intraplantar carrageenan injection significantly induced iNOS protein expression in paws at 24 h. Excavatolide B (15 or 60 mg/kg) treatment subcutaneously 1 h before carrageenan injection significantly inhibited iNOS (**B**) protein expression. The protein expression of β-actin in paw tissue was not significantly different between each group. Quantification values reflect the mean ± SEM of four different experiments. * *p* < 0.05 compared to the control group. ^#^ *p* < 0.05 compared to the carrageenan group.

Western blot analysis was used to assess the effect of excavatolide B on iNOS protein expression in carrageenan-injected paws of rats (Figure 6). The hind paw tissues were collected from the control, carrageenan + vehicle, carrageenan + 15 mg/kg excavatolide B and carrageenan + 60 mg/kg excavatolide B groups at 24 h after carrageenan injection. Figure 6 shows that intraplantar injection of carrageenan significantly increased the iNOS protein expression in the carrageenan group after 24 h compared to the control group. Subcutaneous injection of excavatolide B at two doses, 15 and 60 mg/kg, reduced iNOS protein expression levels to 63.9% ± 7.9% and 30.8% ± 18.1%, respectively, compared to the carrageenan group (100% for iNOS) (Figure 6). Hence, excavatolide B could significantly inhibit iNOS protein expression in paw tissue after carrageenan injection.

## 3. Discussion

### 3.1. Summary

In this study, we successfully isolated excavatolide B from *Briareum excavatum* cultures and found that excavatolide B could significantly downregulate the mRNA expression of the proinflammatory mediators iNOS and COX-2 in LPS-induced murine macrophage cells. In an *in vivo* study, we developed carrageenan-induced paw rat models to assess the anti-inflammatory and analgesic effects of excavatolide B. Administration of excavatolide B (15 or 60 mg/kg) significantly reduced carrageenan-induced paw edema, thermal hyperalgesia and mechanical allodynia after carrageenan injection. Histopathological and Western blot analyses further demonstrated that excavatolide B could reduce carrageenan-induced leukocyte infiltration and iNOS protein expression in paw tissue. These results illustrate that the soft coral-derived compound excavatolide B possesses anti-inflammatory and analgesic activity *in vitro* and *in vivo*, respectively.

### 3.2. The Anti-Inflammatory Effect of Excavatolide B in Vitro and in Vivo Models

Inflammation plays an important role in many diseases, and different cell types are recruited, including monocytes/macrophages, neutrophils, dendritic cells and lymphocytes, to the tissue during the process of inflammation [9,12,37,38]. In the present study, we examined the anti-inflammatory effect of excavatolide B, which is a briarane-type diterpene from soft coral, in *in vitro* and *in vivo* models. Recent reports have indicated that some marine briarane-type diterpenes from soft coral also have anti-inflammatory potential in LPS-induced macrophages [10,11] or peptide (FMLP/CB)-induced human neutrophil cell models [39,40,41]. Moreover, Wei *et al.* has indicated that excavatolide B can reduce the expression of the pro-inflammatory cytokines IL-6 and TNF-α in an LPS-stimulated mouse bone marrow-derived dendritic cell model [32]. Our present results reveal that both iNOS and COX-2 mRNA expressions are inhibited in the LPS-induced murine macrophage model by excavatolide B. Moreover, we also found that excavatolide B dose-dependently inhibited iNOS gene and protein expression *in vitro* and *in vivo*, respectively. Additionally, excavatolide B (25 and 50 μM) reduced COX-2 gene expression at 8 h, but only 50 μM excavatolide B significantly reduced COX-2 protein expression at 18 h. It is known that NO induces COX-2 expression through the transcription factor NF-κB, which is a critical regulator of COX-2 and iNOS expression [25]. The iNOS protein induces massive NO production at the inflammatory site and contributes to COX-2 upregulation in carrageenan-injected rats [26]. Administration of NOS non-selective inhibitor suppressed COX-2 expression in carrageenan-induced inflammation [26]. Our *in vitro* study also demonstrated that COX-2 gene expression is suppressed by excavatolide B. Thus, we suggest that excavatolide B attenuates iNOS expression leading to reduce NO production and results in the downregulation of COX-2 expression in inflammatory processes. Based on the foregoing evidence and present *in vitro* and *in vivo* results, we propose that the anti-inflammatory activity of excavatolide B acts mainly through the downregulation of iNOS.

### 3.3. Effects of Excavatolide B on Carrageenan-Induced Paws in Rats

Carrageenan-induced paw edema in rats is a well-known model for screening potential anti-inflammatory and analgesic agents [29,30,33,42]. The l-arginine-NO pathway has been considered to play an important role in carrageenan-induced pain behavior and inflammatory response [34,43]. iNOS was reported as having an important role in inflammation and produces a massive amount of NO to upregulate blood flow, increase vascular permeability and activate T-lymphocytes in inflammation and pain behavior processes [21,22,23]. Many studies have demonstrated that the massive production of NO via iNOS plays an important role in the development of carrageenan-induced inflammatory nociceptive behaviors [43,44]. Omote *et al.* (2001) and Salvemini *et al.* (1996) have demonstrated the peripheral release of NO in carrageenan-injected tissues [34,45]. iNOS-selective inhibitors inhibit carrageenan-induced nociceptive behavior [43]. The previous study indicated that excavatolide B could reduce the vascular permeability and iNOS protein expression in TPA-induced skin inflammation [32]. In the present study, we also demonstrated that excavatolide B could dose-dependently inhibit iNOS gene and protein expression in *in vitro* and *in vivo* inflammatory models, respectively. Moreover, subcutaneous injection of excavatolide B significantly reduces thermal hyperalgesia, mechanical allodynia, weight bearing deficits and paw edema in carrageenan-induced inflammatory rats. We suggest that the mechanism of excavatolide B on the reduction of pain behaviors in carrageenan-induced rats occurs through the iNOS-NO pathway, since excavatolide B significantly inhibits iNOS expression. 

### 3.4. Histological Analyses of Excavatolide B in Carrageenan-Induced Rats

Previous studies indicated that a large number of neutrophils, monocytes and other inflammatory cells cluster and infiltrate paw tissue after carrageenan injection [36,42,46]. Previous studies indicate that NO could increase vascular permeability leading to inflammatory cell infiltration into inflamed tissue with the release of pro-inflammatory proteins that promote tissue inflammation [13,23]. In the present study, the carrageenan group demonstrated a significantly increased number of neutrophils, monocytes, macrophages and fibroblasts (Figure 5). Our previous study indicated that marine soft coral-derived anti-inflammatory compounds could significantly reduce the number of infiltrating cells in inflammatory animal models [9,30]. According to quantitative analysis, treatment with 15 or 60 mg/kg excavatolide B could significantly reduce the number of infiltrating cells in carrageenan-induced paw edema in rats (Figure 5E–H). Previous studies have also shown that excavatolide B can improve vascular permeability in TPA-induced skin inflammation [32]. Our data also show that iNOS protein expression was inhibited in carrageenan-treated paw tissues after 15 or 60 mg/kg excavatolide B administration. Thus, excavatolide B could reduce the infiltration of cells, such as neutrophils, monocytes and macrophages, through improved vascular permeability in carrageenan-induced rat models.

### 3.5. Coral Aquaculture for Drug Discovery

Coral reefs are among the most productive marine ecosystems, and there are over 3,000 new compounds that have been isolated from corals in the past two decades [1,4,5]. Currently, the search for marine natural products typically depends on wild specimens, and an extensive collection of corals for drug development would disrupt the ecological processes of ecosystems [47,48]. This is a critical limitation for the development of new marine-derived drugs. In the present study, excavatolide B is the target metabolite, which is stable when isolated from or produced by the cultured coral *Briareum excavatum*. Aquaculture of coral may overcome the aforementioned limitation and may prompt more drug discovery research [5]. Moreover, coral aquaculture is not only used for marine ecosystem restoration, but it could also be used continuously to produce target compounds by using homogenous environmental conditions [5]. In our previous research, we have successfully studied some compounds *in vitro* and *in vivo* from cultured coral, such as 11-dehydrosinulariolide, sinularin and 11-*epi*-sinulariolide acetate, which can also be isolated from wild coral [8,9,30,49]. In the present study, excavatolide B can be substantial and stable when isolated from cultured *Briareum excavatum*, which yields about 0.76 g/kg (wet weight), and the yield of wild-type *Briareum excavatum* is about 0.24 g/kg [31]. Coral aquaculture could also be used for new compound discovery [50,51]. Hence, coral aquaculture has potential and may be essential for drug development.

## 4. Experimental Section

### 4.1. Preparation of Excavatolide B

In the present study, excavatolide B (1*R*, 2*S*, 4*S*, 4a*R*, 5*S*, 6*S*, 8*Z*, 9a*S*, 12a*S*, 13*R*, 13a*S*)-4,5,13-triacetoxy-2-hydroxy-1,4a,8,11a-tetramethyl-11-oxo-2,3,4,4a,5,6,7,9a,11,11a,13,13adodecahydro-1H benzo[4,5]cyclodeca[1,2-b]oxireno[c]furan-6-yl butyrate (Figure 1A) was isolated from Formosan gorgonian *Briareum excavatum* (wet weight of 2.8 kg), which was collected in 0.6-ton cultivating tanks in National Museum of Marine Biology & Aquarium (NMMBA), Taiwan. Briefly, corals were minced and exhaustively extracted with methanol and dichloromethane (1:1). The organic extract was partitioned into H_2_O and ethyl acetate layers, and the ethyl acetate layer was separated further over a normal phase silica gel by column chromatography eluted with *n*-hexane, ethyl acetate and methanol to yield 30 fractions. Fractions 13–16 was separated using a normal phase silica gel elution with n-hexane-ethyl acetate to yield a subfraction and then further purified by a normal phase silica gel elution with *n*-hexane-ethyl acetate (80:1) to acquire the pure compound, excavatolide B (2.13 g). The structure of the compound was confirmed by nuclear magnetic resonance spectroscopy. The purity (>98%) of excavatolide B was evaluated and verified by 1H-NMR and 13C-NMR spectra (Varian Mercury Plus 400 FT-NMR at 400 MHz). Furthermore, the structure was also compared to that of natural compounds derived from soft coral, as described previously [31].

### 4.2. Cell Culture

*In vitro* anti-inflammatory activity was evaluated according to previous studies with modifications [9,30]. Murine RAW 264.7 macrophages were obtained from the American Type Culture Collection (ATCC, No TIB-71) and cultured in Dulbecco’s Modified Eagle Medium (DMEM, 10% heat-inactivated FBS, 1 mM pyruvate, 2 mM glutamine, 4.5 g/L glucose, 50 μg/mL streptomycin and 50 U/mL penicillin) at 37 °C in a humidified incubator under standard conditions with 5% CO_2_:95% air. The inflammatory response in RAW 264.7 cells was assessed by challenging cells in medium containing only 10 ng/mL LPS (Sigma-Aldrich, St. Louis, MO, USA) alone. For the anti-inflammatory activity assay, excavatolide B (1, 10, 25 and 50 μM) was added to cells 10 min before the LPS challenge. We obtained cell pellets by washing with ice-cold phosphate-buffered saline (PBS, 137 mM NaCl, 2.68 mM KCl, 10 mM Na_2_HPO_4_, 1.76 mM KH_2_PO_4_, pH = 7.2) and centrifugation at 3000 rpm for 10 min at 4 °C, and then, they were stored at −80 °C until analyzed.

### 4.3. Cell Viability

Cell viability was determined after treatment with Alamar Blue (Invitrogen, Carlsbad, CA, USA), the tetrazolium dye that is reduced by living cells to fluorescent products. This assay is similar in principle to the cell viability assay using 3-(4,5-dimethyldiazol-2-yl)-2,5-diphenyltetrazolium bromide and has been validated as an accurate measure of the survival of RAW 264.7 macrophage cells [36,52]. In cell culture experiments, excavatolide B was dissolved in 100% dimethyl sulfoxide (DMSO) (clear). The final concentration of DMSO in the culture medium was 0.1%. The control in the final culture medium was 0.1% DMSO.

### 4.4. Western Blot Analysis for iNOS, COX-2 and β-actin

Western blotting was performed as described in our previous studies [9,36]. Cell pellets were collected from centrifuged tubes after 18 h and lysed in 4 °C lysis buffer (50 mM Tris, pH 7.5, 150 mM NaCl, 1% Triton X-100, 100 μg/mL phenylmethylsulfonyl fluoride (PMSF), 1 μg/mL aprotinin) and centrifugated at 14,000 rpm for 30 min at 4 °C. The supernatant was collected from the pellet and the protein concentrations were determined by the DC Protein Assay kit (Bio-Rad, Hercules, CA, USA) [53]. An equal volume of sample buffer (2% sodium dodecyl sulfate (SDS), 10% glycerol, 0.1% bromophenol blue, 2% 2-mercaptoethanol and 50 mM Tris–HCl, pH 7.2) was added to the sample, and we electrophoresed the protein by tricine SDS-polyacrylamide (7% or 10%) gel at 80 V for 150 min. Proteins were transferred to polyvinylidene difluoride (PVDF) membranes (Immobilon-P; pore size, 0.45 μM; Millipore, Bedford, MA, USA) at 135 mA 16–18 h at 4 °C in transfer buffer (50 mM Tris–HCl, 380 mM glycine, 1% SDS, 20% methanol). Membranes were blocked for 60 min at room temperature with 5% non-fat dry milk in Tris-buffered saline with Tween 20 (TTBS; 0.1% Tween 20, 20 mM Tris–HCl, pH 7.4, 137 mM NaCl) and then were incubated at 4 °C overnight with primary antibodies against iNOS (polyclonal antibody; 1:1500 dilution; BD Pharmingen, San Diego, CA, USA), COX-2 (1:2000, Cayman Chemical, Ann Arbor, MA, USA; Catalog No. 160106; polyclonal antibody) and β-actin (monoclonal antibody; 1:1500 dilution; Sigma-Aldrich, St. Louis, MO, USA) proteins. The iNOS, COX-2, and β-actin antibodies recognized bands at ~135, ~72 and ~45 kDa, respectively. The immunoreactive bands were visualized by enhanced chemiluminescence (Millipore, Billerica, MA, USA) and the Biochemi Imaging System, and relative densitometric quantification was performed using LabWorks 6.2 software (UVP, Upland, CA, USA).

### 4.5. Real-Time PCR Analysis for iNOS and COX-2 mRNA

Real time quantitative polymerase chain reaction (qPCR) was performed according to methods described by Livak and Schmittgen, (2001) and De Gois *et al.* (2005) with modifications [54,55]. Cell pellets were collected from centrifuged tubes after 8 h, and total RNA was isolated using TRIzol^®^ RNA Isolation Reagents (Catalog No. 15596-026, Life Technologies, Van Allen Way, Carlsbad, CA, USA) according to the manufacturer’s instructions. After centrifugation at 3000 rpm for 8 min at 4 °C, total RNA was obtained and transcribed using the iScript cDNA synthesis kit (Bio-Rad, Hercules, CA, USA). The reactions were performed in duplicate with 0.5 μL of each primer (0.2 μM final concentration), 25 μL of iQ SYBR Green Supermix (Bio-Rad, Hercules, CA, USA; 100 mM KCl, 40 mM Tris-HCl, pH 8.4, 0.4 mM of each dNTP, iTaq DNA polymerase, 50 units/mL, 6 mM MgCl_2_, SYBR Green I, 20 nM fluorescein and stabilizer) and 2.5 μL of template in a 50-μL total volume. The PCR cycle conditions were 95 °C for 10 min, 40 cycle of 95 °C for 15 s and 60 °C for 1 min. A melting curve analysis was performed at the end of each experiment to verify that a single product per primer pair was amplified. The amplification and analysis were performed using a CFX96 TouchTM Real-time PCR Detection System (Bio-Rad, Hercules, CA, USA). Results were compared using the relative cycle threshold (CT) method. The fold increase or decrease was determined relative to a blank control after normalizing to a housekeeping gene (GAPDH) using 2^−ΔΔ*C*T^ [54,55]. The real-time PCR oligonucleotide primers used for genotyping are as follows: iNOS (forward), 5′-GCTGTTAGAGACACTTCTGAG-3′; iNOS (reverse), 5′-CACTTTGGTAGGATTTGACTTTG-3′; COX-2 (forward), 5′-CTCTGAACTATGGTGTGAACAATC-3′; COX-2 (reverse), 5′-GTCAGTCTTTATAACATGCTTGG G-3′; β-actin (forward), 5′-GCTTCTTTGCAGCTCCTTC-3′; β-actin (reverse), 5′-GACCAGCGCAGCGATATC-3′. 

### 4.6. Preparation of Animals

Wistar rats (250–285 g) were received from LASCO Inc. (Taipei, Taiwan) and were used only in this experiment. Rats were maintained in Plexiglas cages and given free access to food and water in a temperature-controlled (24 ± 1°C), 12-h light/dark cycle room. Excavatolide B and 1.5% carrageenan injections were performed under 2.5% isoflurane (Panion & BF Biotech Inc., Taoyuan, Taiwan, Catalog No. 08547) anesthesia. All animal experiments followed the Guiding Principles in the Care and Use of Animals of the American Physiology Society and were approved by the institutional animal care and use committee of National Sun Yat-sen University (Dated 4 March 2011, Study No. 10003).

### 4.7. Animal Experimental Design

Male Wistar rats (*n* = 30) were randomly divided into six groups: control (0.5 mL saline with 20% DMSO, *n* = 6), 1.5% carrageenan + vehicle (*n* = 6), 1.5% carrageenan + 15 mg/kg excavatolide B (*n* = 6), 1.5% carrageenan + 60 mg/kg excavatolide B (*n* = 6), 1.5% carrageenan + 5 mg/kg indomethacin (*n* = 6) and 60 mg/kg excavatolide B. The method of carrageenan injection was modified from our previous study [29,30]. The control was 0.5 mL 20% DMSO. Rats were induced with paw edema and pain behaviors by 1.5% sterile lambda carrageenan (Sigma-Aldrich Inc., St. Louis, MO, USA) in 100 μL saline intraplantar injection into the right hind paw. Rats received excavatolide B (15 or 60 mg/kg dissolved in 0.5 mL 20% DMSO) by subcutaneous injection 1 h before carrageenan injection. 

### 4.8. Measurement of Paw Edema

Paw edema was measured as a marker of tissue inflammation. Rats were anesthetized with 2.5% isoflurane, and then paw edema was measured using a Paw Volume Meter (plethysmometer, Diagnostic & Research Instruments Co., Ltd., Singa, Taoyuan, Taiwan) after injection with 1.5% carrageenan and excavatolide B treatment. The change in paw edema was calculated by subtracting the initial paw edema (basal value) from the value measured at 1, 2, 3, 4, 5, 6, 9, 12 and 24 h after carrageenan injection.

### 4.9. Thermal Hyperalgesia

Thermal hyperalgesia was evaluated by placing the hind paw on a radiant heat source at a low intensity heat and measuring the paw withdrawal latency with the cut-off time set at 30 s (active intensity = 25) using an IITC analgesiometer (IITC Inc., Woodland Hills, CA, USA). The paw withdrawal latency was assessed as described previously by Hargreaves *et al.* (1988) and our previous study as the average of three measurements per paw [30,56]. The hind paw plantar test was measured at 1, 2, 3, 4, 5, 6, 9, 12 and 24 h after carrageenan injection.

### 4.10. Mechanical Allodynia

To assess mechanical allodynia, the hind paw withdrawal thresholds were measured using calibrated von Frey filaments (Stoelting, Wood Dale, IL, USA). Rats were placed in compartments of brown plastic cages on top of an elevated metal mesh floor, permitting easy access to the paws. The von Frey filaments of logarithmically incremental stiffness were applied to the mid-plantar region of the rat hind paw from below the mesh floor using Chaplan’s “up-down” method, involving the use of alternate larger and smaller fibers to determine the closest filament to the threshold of pain response (licking or withdrawal), as described previously by Chaplan *et al.* (2011) and by our previous study [29,30,57].

### 4.11. Weight-Bearing Distribution Test

The effect of excavatolide B on weight-bearing capacity with the right and left paw was measured using a Dual Channel Weight Averager (Diagnostic & Research Instruments Co., Ltd., Singa, Taoyuan, Taiwan), which independently detects the weight borne on each paw. Rats were placed in a brown plastic chamber that is designed to have each hind paw resting on a separate weight sensor, which records the average value of the animal’s body weight distribution on each paw every three seconds. Changes in hind paw weight distribution (in grams) are shown as the difference between the affected limb (the right hind paw) and the normal limb (the left hind paw), which were measured at the same time point [58,59].

### 4.12. Western Blot Analysis for iNOS in Carrageenan-Induced Paw Edema

After the last paw edema measurement (24 h after carrageenan injection), the animals were sacrificed, and paw samples were collected for Western blotting. The paw tissues from the paws of the control, 1.5% carrageenan + vehicle, 1.5% carrageenan + 15 mg/kg excavatolide B and 1.5% carrageenan + 60 mg/kg excavatolide B groups were collected and washed with PBS and homogenized in lysis buffer using a Polytron homogenizer (Precellys^®^24, tissue homogenizer; Bertin Technologies, Aix En Provence, Rockville, MD, USA). They were then ultracentrifuged at 65,000 rpm for 1 h at 4 °C, and the supernatant was measured using a DC protein assay kit (Bio-Rad, Hercules, CA, USA). Protein samples were then used for Western blot analysis of iNOS and β-actin.

### 4.13. Histopathological Analysis

For the histopathological examination, we modified the method from our previous study [29,30]. Rats were sacrificed after perfusion with PBS and 4% paraformaldehyde at 24 h after carrageenan injection, and the paw tissues were fixed in 10% neutral formalin for 3–4 days. The paws were decalcified with 12.5% ethylenediaminetetraacetic acid (EDTA) in 10% neutral formalin for 4 weeks and sectioned on the sagittal plane through the center of samples. The specimens were dehydrated in a graded series of alcohol and embedded with paraffin, and 2-μm sections were prepared for hematoxylin and eosin staining to assess the general and pathological changes in morphology using microscopic examination by light microscopy (DM 6000B, Leica Inc. Wetzlar, Germany) with a microscope digital image output system (idea SPOT, Diagnostic Instruments Inc., Steriling Heights, MI, USA). We evaluated whether excavatolide B attenuated the inflammation in carrageenan-induced rats while reducing the number of neutrophils, macrophages, monocytes and fibroblasts in paw tissue in a high power field (400×) [9,36,60,61]. 

### 4.14. Statistical Analysis

All data are presented as the mean ± SEM. For the immunoreactivity data, the intensity of each test band is expressed as the integrated optical density (IOD), calculated with respect to the average optical density of the corresponding control (LPS or alone) band. For statistical analysis, all of the data were analyzed by a one-way analysis of variance (ANOVA), followed by the Duncan’s method for multiple comparisons (Systat Software, San Jose, CA, USA). We defin ed a significant difference as *p* < 0.05. 

## 5. Conclusions

In this study, we isolated and purified excavatolide B from the soft coral *Briareum excavatum*. Our *in vitro* study revealed that excavatolide B significantly inhibited the gene expression of the pro-inflammatory proteins iNOS and COX-2 in LPS-challenged murine macrophages. The *in vivo* study revealed that treatment with 15 or 60 mg/kg excavatolide B could significantly reduce mechanical allodynia, thermal hyperalgesia, weight-bearing deficits and paw edema in carrageenan-induced inflammatory rats. Moreover, using histological analysis, we found that excavatolide B could improve the cluster and infiltration of neutrophils, monocytes, macrophages and fibroblasts and reduce the expression of the pro-inflammatory protein iNOS in carrageenan-induced inflammatory rats. We conclude that excavatolide B may reduce the infiltration of inflammatory cells and iNOS protein expression to ameliorate the pain behavior and inflammatory response in carrageenan-induced inflammatory rats. Hence, the soft coral-derived compound excavatolide B may serve as a useful therapeutic agent for the treatment of acute inflammation.
